# Mechanistic study of the solubilization effect of basic amino acids on a poorly water-soluble drug†

**DOI:** 10.1039/d2ra02870k

**Published:** 2022-06-29

**Authors:** Mohammed Suleiman Alsalhi, Paul G. Royall, Ka Lung Andrew Chan

**Affiliations:** Institute of Pharmaceutical Science, King's College London SE1 9NH UK ka_lung.chan@kcl.ac.uk

## Abstract

Amino acids have shown promising abilities to form complexes with poorly water-soluble drugs and improve their physicochemical properties for a better dissolution profile through molecular interactions. Salt formation *via* ionization between acidic drugs and basic amino acids is known as the major contributor to solubility enhancement. However, the mechanism of solubility enhancement due to non-ionic interactions, which is less pH-dependent, remains unclear. The aim of this study is to evaluate non-ionic interactions between a model acidic drug, indomethacin (IND), and basic amino acids, arginine, lysine and histidine, in water. At low concentrations of amino acids, IND–arginine and IND–lysine complexes have shown a linear relationship (A_L_-type phase solubility diagram) between IND solubility and amino acid concentration, producing ∼1 : 1 stoichiometry of drug-amino acid complexes as expected due to the strong electrostatic interactions. However, IND–histidine complexes have shown a nonlinear relationship with lower improvement in IND solubility due to the weaker electrostatic interactions when compared to arginine and lysine. Interestingly, the results have also shown that at high arginine concentrations, the linearity was lost between IND solubility and amino acid concentration with a negative diversion from linearity, following the type-A_N_ phase solubility. This is indicative that the electrostatic interaction is being interrupted by non-electrostatic interactions, as seen with histidine. The IND–lysine complex, on the other hand, showed a complex curvature phase solubility diagram (type B_S_) as lysine self-assembles and polymerizes at higher concentrations. The freeze-dried drug–amino acid solids were further characterized using thermal analysis and infrared spectroscopy, with results showing the involvement of weak non-ionic interactions. This study shows that the solubility improvement of an insoluble drug in the presence of basic amino acids was due to both non-ionic and ionic interactions.

## Abstract 2

Amino acids have shown promising abilities to form complexes with poorly water-soluble drugs and improve their physicochemical properties for a better dissolution profile through molecular interactions.

## Graphical abstract

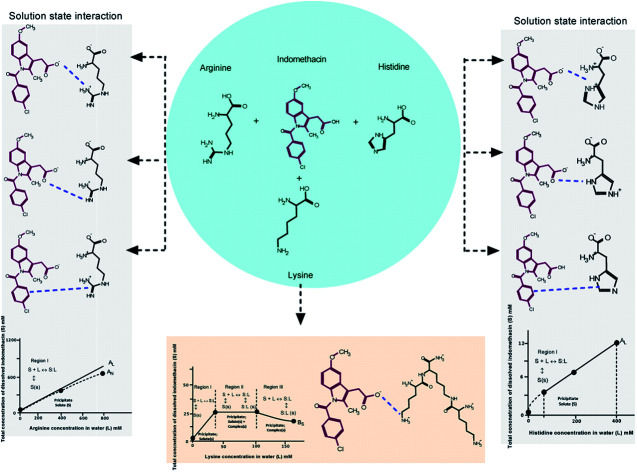

## Introduction

Most oral active pharmaceutical ingredients (API) suffer from low water solubility. These drugs are classified as class II or IV in the biopharmaceutics classification system (BCS).^1^ Improving solubility is one of the most important steps in pharmaceutical development. Various formulation strategies have been developed to optimize and overcome limitations associated with poor aqueous solubility.^2,3^ Salt formation is considered as a primary approach to enhance the aqueous solubility and dissolution profile of weakly acidic or basic drugs.^4^ Amino acids recently have gained considerable interest as low molecular weight counterions to produce the salt form of the poorly water-soluble drugs.^5–11^ The salt form of acidic or basic drugs shows a significant improvement in aqueous solubility and dissolution rate compared to their corresponding free basic or acidic forms.^12–15^ The physicochemical properties of the salt former play a significant role in the optimal salt form for drug candidates.^16^

The use of amino acids as counterion co-formers to improve the solubility of drugs recently has been concentrated on producing co-crystals or co-amorphous systems. These systems are typically prepared by mechanical activation, such as ball milling, while others are based on solvent evaporation from organic solvent, hot-melt extrusion or melt-quench techniques.^5–12,14,17–22^ Inconsistent outcomes by using different preparation techniques could be seen for acidic drugs with basic amino acids such as lysine and arginine. For example, dry ball milling was shown to be able to convert indomethacin and naproxen into co-amorphous in the presence of these amino acids, but not through liquid-assisted grinding.^8,20,23^ Interestingly, indomethacin became co-amorphous with histidine, a weak base, upon spray drying from an organic solvent,^9^ but not after ball milling or aqueous freeze-drying.^7,24^ Work by ElShaer *et al.*^7^ have illustrated the saturated solubility of indomethacin in the presence of these basic amino acids and the results showed an improvement in drug solubility. However, freeze-dried indomethacin–histidine did not form co-amorphous salt, suggesting that the methods used for the preparation have significant impacts on the properties of the final products. Indeed, the effect of different production techniques on the behavior of these salt systems was not comprehensively investigated. Another reason for the inconsistency could have resulted from the degradation of the original material and hence the generation of impurities. Impurities may be present in a small concentration, but can have a significant effect on physical processes.^25^ Previous attempts to produce the co-amorphous salt systems involve relatively aggressive processes, which could lead to degradation. The approaches used in this paper are low energy processes and therefore do not degrade the drug or amino acids. Wostry^26^ and ElShaer^7^ produced freeze-dried co-amorphous salt of naproxen and indomethacin with arginine and lysine but not with histidine. Moreover, limited works have been published to optimize the ratio between indomethacin and amino acids, which is important for formulation development.^19,27,28^ With the absence of this information, it has been assumed that a 1 : 1 drug: co-former is the most preferable mole ratio. The role of basic amino acids at the molecular level for the enhanced aqueous solubility of insoluble drugs was not yet fully understood.

Among counterion co-formers reported in the literature, amino acids have the advantage of being generally regarded as safe (GRAS) natural compounds with low molecular weights, providing the potential to improve drug loading compared to polymers.^29^ Amino acid consists of a carboxylic acid group with an aliphatic primary amino group in the alpha (α) carbon and an R-group attachment (the side chain). The side chain can be acidic, basic, or neutral. In a buffer solution of pH 7.4 the carboxyl group will be deprotonated to carboxylate (anion) and the amino group will be protonated to ammonium (cation), forming a soluble zwitterions compound, ^+^H_3_N–CHR–COO^−^_(aq)_, stabilized by electrostatic interactions and hydrogen bonds. A wide scope of functional groups at the side chain can contribute to the different physicochemical properties and the possibility to produce various complexations with poorly water-soluble drugs. We have demonstrated that neutral amino acids have a hydrotropic effect and thus increase the solubility of indomethacin through non-ionic interactions.^30^ Whilst the main contributor on solubility improvement for acidic drugs in the presence of basic amino acids is ionic interaction, the influence of non-ionic interactions between basic amino acids and acidic drugs has not been clearly identified. Non-ionic interactions are important as they are not affected by pH and could be important in the prevention of disproportionation.

**Fig. 1:**
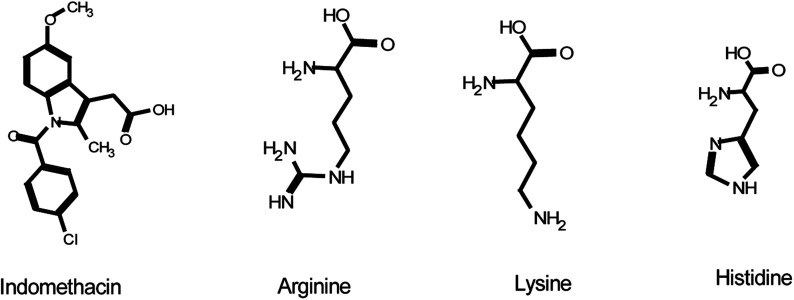
Chemical structure of indomethacin, arginine, lysine and histidine.

The aim of this study is to evaluate the potential non-ionic interactions between the basic amino acids, arginine, lysine, and histidine, and the weakly acidic model drug, indomethacin in the liquid and solid states. The chemical structures are shown in Fig. 1. Indomethacin is a practically insoluble BCS class II API. It is prescribed for inflammation, analgesia and rheumatoid arthritis.^31^ Phase solubility studies were performed at 30 ± 2 °C and 45 ± 2 °C using UV/Vis spectroscopic analytical technique. The solubility profile was constructed to assess the effect of the amino acids on the solubility of the drug. The solubility studies, as defined by Higuchi and Connors, have been conducted to explore and understand the chemistry of such system, emphasizing on the nature of molecular interactions between compounds under defined conditions such as temperature, pressure and constant volume.^32^ The molecular interactions can be described in a quantitative manner by knowing the stoichiometry of the equilibrium. A phase diagram is constructed by plotting, on the vertical axis, the total concentration of drug found in the solution phase against the known concentration of amino acids added to the system. In general, two types of phase diagrams (type A or type B) could be observed. For type A, the solubility of drug is increased linearly by the presence of amino acids, known as A_L,_ while positive or negative diversion form linearity are known as A_P_ and A_N_, respectively. For B type however, the solubility of drug is strongly affected by the presence of amino acids, producing complex curvature either B_S_ or B_I_. The solid-state of drug–amino acid systems after freeze-drying was further investigated through Fourier transform infrared spectroscopy, UV-Vis spectroscopy, thermogravimetric analysis and differential scanning calorimetry.

## Experimental

### Materials

Indomethacin (IND, γ polymorph), l-histidine (HIS), l-lysine (LYS), l-isoleucine (ISO), glycine (GLY); 99% purity, were purchased from Alfa Aesar (Heysham, England). l-arginine (ARG); ≥99.5% purity, was purchased from Sigma Aldrich (Nottingham, United Kingdom). All powders were used as received from the supplier, and properties of the raw chemicals are described in Table 1. Ultrapure water was produced from a Pure Lab Ultra-System (London, United Kingdom), hydrochloric acid 32% and sodium hydroxide; ≥97% purity, were supplied by Sigma Aldrich (Poole, United Kingdom) and VWR international (Leicestershire, United Kingdom), respectively. A pH meter (OXOID limited, England) was calibrated with standard buffer solutions: phosphate buffers; pH 7 and 4 ± 0.2 at 25 ± 1 °C and used to determine the pH of the solutions. A temperature probe (YC-747UD data logger thermometer) was used to accurately determine the solution temperature (precision 0.1 °C).

**Table 1:** Properties of γ indomethacin and amino acids; molar mass, density, hydrogen bond acceptor and donor, aqueous solubility, acid dissociation constant of the side chain (p*K*_a_), isoelectric point (pI), partition coefficient (log *p*) and *λ*_max_ UV absorbance at pH 7 (ref. 34)

Chemical substance	Molar mass (g mol^−1^)	Density (g cm^−3^)	Hydrogen donor	Hydrogen acceptor	Water solubility (g/100 mL) at 25 °C	p*K*_a_ (side chain)	pI	log *p*	UV abs (*λ*_max_ nm) at pH 7
Indomethacin	357.79	1.38	1	4	0.009	4.42	—	4.05	264–320
Arginine	174.20	1.33	4	4	18.20	12.48	10.76	−4.20	—
Lysine	146.19	1.24	3	4	Very soluble	10.53	9.47	−3.05	—
Histidine	155.15	1.41	3	4	4.20	6.04	7.59	−3.32	211 (ref. 33)
Isoleucine	133.17	1.29	2	3	0.41	—	6.04	−1.70	—
Glycine	75.07	1.16	2	3	24.99	—	5.97	−3.21	—

### Methods

#### UV-Vis spectrophotometric analysis

The UV-Vis spectra of the model drug in arginine, lysine and histidine solution at a pH range of 2 to 12 were measured using a double beam PerkinElmer Lambda 2 UV-Vis spectrophotometer. Spectra were collected using the UV Lambda software over the wavelength range of 400 nm–200 nm. The same solvent without the drug/amino acid was used as the reference. Samples were analyzed at identical conditions: slit width 1 nm, bandwidth 0.5 nm, pair cuvette cells 1 cm.

#### Preparation of stock solution and calibration curve

A standard calibration curve was obtained by measuring solutions of concentration range from 0.5 μg mL^−1^ to 14 μg mL^−1^ prepared by serial dilution from a stock solution of indomethacin (20 μg mL^−1^ in water (pH of 6.3 ± 1). Stock solutions of basic arginine, lysine and histidine amino acids were prepared at 2500 μg mL^−1^ in pure water. Four solutions of indomethacin of concentration range from 0.5 μg mL^−1^ to 12 μg mL^−1^ in 250 μg mL^−1^ of arginine, lysine and histidine solutions at pH 10.3, 10 and 7.5 were prepared respectively. Samples were analyzed spectrophotometrically in triplicate at 260 nm and 320 nm.

#### Validation of UV method

The method was validated for accuracy, precision, limit of detection (LOD), limit of quantitation (LOQ), linearity and range. The linearity of calibration curves was determined over the concentration range of 0.5–14 μg mL^−1^ and 0–12 μg mL^−1^ for traditional and standard calibrations, respectively. Inter-day precision was carried out by measuring three different concentrations of indomethacin on three different days in a week by calculating the relative standard deviation (RSD%) of absorbance. Repeatability was assessed by running the same concentration of drug three times. The limit of detection and quantification (LOD and LOQ) were calculated *via* formulas: LOD = (3.3 × STD/*S*) and LOQ = (10 × STD/*S*) where STD refers to the standard deviation and *S* is the slope of the calibration curve.^35^

#### pH-solubility study

The solubility of indomethacin in water at fixed pH 1.2 and 6.3 was performed with the shake flask method at constant volume, proposed by Higuchi and Connors at a constant temperature of 25 ± 2 °C and 30 ± 2 °C.^32^ Excess indomethacin was added to 10 mL water, each was performed in triplicate. The samples were bath sonicated for 2 minutes and stirred for 24 hours until equilibrium was established. The samples were then filtered using a membrane filter (syringe filter, 0.45 μm sterile). The filtered solutions were diluted within the range of calibration curves and analyzed spectrophotometrically at 260 nm.

#### Preparation of phase solubility diagrams

Phase solubility diagrams of indomethacin in basic arginine, lysine and histidine amino acid solutions were conducted with the shake flask method at different temperatures (25 ± 2 °C or 30 ± 2 °C) as described above. After equilibration, the mixtures were centrifuged at 3750 rpm for 10 minutes (Beckman Coulter Allegra X-12 Series). Two layers (liquid supernatant and solid precipitation) were obtained as shown in the exemplary diagram (ESI Fig. S1†). The supernatant was filtered *via* a membrane filter (syringe filter, 0.45 μm sterile). The solutions were then diluted within the calibration curve range and analyzed spectrophotometrically at 260 nm. The precipitates (solid phase) were filtered again using filter paper (Whatman Grade 3). The solid phase was dried in an oven at 70 °C for 30 minutes, then examined using FTIR. The difference spectra were generated by subtracting the spectrum of the precipitate from the spectrum of the pure γ-indomethacin.

#### Complexation efficiency of drug–amino acid

The complexation or solubilizing efficiency (CE)^36^ was calculated from the slope of the linear drug–amino acid phase solubility (PS) diagram using the following eqn (1):1
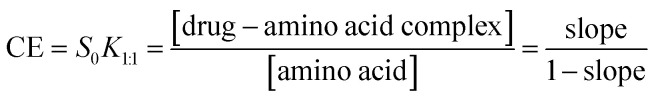
where *K*_1:1_ indicates to the stability constant, which is used to compare the affinity of drug to different amino acids. *S*_0_ refers to the intrinsic solubility, [drug–amino acid complex] is the concentration of dissolved drug–amino acid complex, [amino acid] is the concentration of dissolved free amino acid and slope is the slope of the phase solubility diagram.

#### Physical and chemical stability tests

Saturated indomethacin in solutions of arginine, histidine and lysine (5 mg mL^−1^, 5 mg mL^−1^ and 2 mg mL^−1^, respectively) at pH 7 and room temperature (25 ± 1 °C) were visually inspected for physical stability over one week against a black and a white background to determine any their physical appearances such as colour, turbidity and precipitation. Indomethacin stability (10 μg mL^−1^) in these amino acid solutions at concentration of 2.5 mg mL^−1^, which has pH of 10.3 and 10 and 7.6 respectively, were also examined. The results were recorded for samples stored over a week at ambient temperature (25 ± 1 °C). Chemical stability was confirmed by measuring the UV absorbance at 320 nm.

#### Preparation of hydrotropic solutions

Hydrotropic solubilization of arginine, isoleucine, glycine and histidine amino acids solutions over concentrations ranging from 0.1 M to 1.6 M at controlled pH 1.2 and 7 were prepared in triplicate. The pH of the solutions was adjusted by adding 0.1 M hydrochloric acid and monitored with a pH meter (OXOID limited, England). Excess indomethacin was then added to the amino acid solutions. The suspension was stirred with a magnetic stirrer for 24 hours at 30 ± 2 °C or 45 ± 2 °C until it is equilibrated followed by adjusting the pH again. The samples were then filtered *via* a membrane filter (syringe filter, 0.45 μm sterile). The resulting filtrate was diluted to determine the saturated solubility of indomethacin in amino acid solutions *via* UV spectrometry at 260 nm.

#### Preparation of solution of indomethacin-basic amino acids for freeze-drying

Saturated indomethacin in amino acids solutions was prepared according to the phase solubility studies as described above. The resulting filtrates were frozen for 24 hours at −80 °C and then freeze-dried for 48 hours (LTE Scientific Lyotrap Freeze Dryer, Great Britain, Greenfield, Oldham) with the system temperature of −51 ± 2 °C, pressure of around 0.1 mbar and the vacuum pump operated at a flow rate of 21 m^3^ h^−1^. The freeze-dried samples were then transferred into a plastic vacuum desiccator containing phosphorous pentoxide desiccant (P_2_O_5_) at ambient temperature (22 ± 2 °C). Indomethacin–sodium salt was also produced for comparison purposes.

#### UV-VIS spectrophotometry

Indomethacin–basic amino acids systems were examined using UV-Vis for their drug content and chemical stability after freeze-drying. The freeze-dried samples were prepared by dissolving 10 mg freeze-dried materials in water in a 10 mL volumetric flask. The solutions were then diluted to concentrations that fall within the calibration curve and analyzed at 320 nm. The percentage drug content^37^ was calculated by eqn (2)2
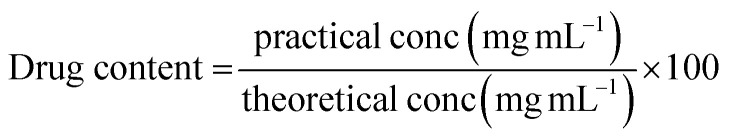


#### Fourier transform infrared spectroscopy (FTIR)

Infrared spectra were collected using an FT-IR spectrometer (Spectrum One, PerkinElmer, Seer Green, UK) with a diamond attenuated total reflectance (ATR) accessory (Golden Gate™, Specac Ltd, UK) for the freeze-dried indomethacin–amino acids measurements. Samples were collected with the Spectrum 10 software over a range of frequency 4000–400 cm^−1^ and an accumulation of 64 scans with 4 cm^−1^ resolutions. A background spectrum was collected with a clean ATR surface. Reference amorphous indomethacin was obtained by quench cooling from the melting temperature (162 ± 2 °C) to room temperature.

#### Differential scanning calorimetry (DSC)

DSC was performed using a Q20 instrument V24.7 build 119 thermal analysis system with an intercooler system (Elstree, United Kingdom). The DSC system was calibrated and validated using indium (average melting temperature 156.36 ± 0.03 °C and enthalpy 29.81 ± 0.05 J g^−1^). Powder samples of 1 to 5 mg were weighed in T-zero aluminum pans with T-zero lids (TA Instruments – A Division of Waters Ltd, Centennial Avenue, Elstree, Hertfordshire, United Kingdom). The pure amino acids as received and physical mixtures of drug–amino acids 1 : 1 molar ratio samples were conducted under a flow of 50 mL min^−1^ nitrogen purge gas, heated to 300 °C with a heating rate of 5 °C min^−1^, except for the pure indomethacin as received and the freeze dried complexes indomethacin–amino acids, where these samples were heated to 200 °C due to instability issue above the melting point of indomethacin.^38^ All results were analyzed using the TRIOS software for the phases transitions to determine the glass transition temperature (*T*_g_) and melting point (*T*_m_).

Indomethacin (as received) was analyzed in two heating cycles to produce an amorphous form. The first cycle was to measure the *T*_m_ of the crystalline form and the second cycle was for obtaining the *T*_g_ of amorphous indomethacin. The freeze-dried complexes and crystalline arginine, histidine and lysine were undergone one heating cycle to determine the *T*_g_ or *T*_m_. The *T*_g_ described by Gordon–Taylor^39^ was determined for drug–basic amino acids binary systems using the given equations below.

The Gordon–Taylor equation from composite glasses:3
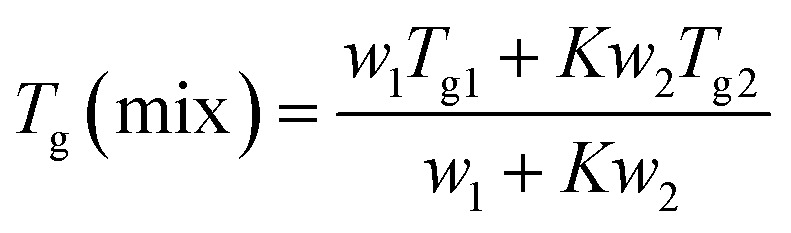


*T*_g_(mix) is the glass transition temperature of a binary mixture, *w*_1_ and *w*_2_ are the weight fraction and *T*_g1_ and *T*_g2_ are the glass transitions temperature of individual compound in the mixture, *K* is typically considered an empirical constant:4
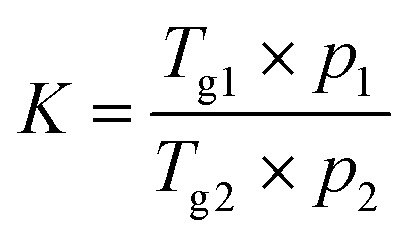
where *p*_1_ and *p*_2_ are the powder densities of the single amorphous components. The crystalline densities of compounds (*p*_IND_ = 1.379 g cm^−3^*p*_ARG_ = 1.325 g cm^−3^, *p*_HIS_ = 1.412 g cm^−3^ and *p*_LYS_ = 1.237 g cm^−3^) were used as approximation because small differences in density were reported between crystalline and amorphous form of small molecular compound.^9^

#### Thermogravimetric analysis (TGA)

The thermal stability analysis of the samples was conducted by using a thermogravimetric analyzer (TA instrument Q500 TGA). The system was calibrated using an empty platinum pan. 5–10 mg of pure crystalline drug powder, amino acids, physical mixtures drug–amino acids 1 : 1 molar ratio or freeze-dried drug–amino acids were continuously weighed in the open platinum pan under nitrogen from 30 °C to 300 °C at a heating rate of 10 °C min^−1^. All results were analyzed using the TRIOS analysis software.

## Results

### UV-VIS analysis

The UV-Vis spectra of indomethacin in aqueous solutions of pH between 1 and 13, and in the presence of amino acids were investigated. The UV spectra of indomethacin have shown large differences when measured in pH 1 and pH 6–8 due to the p*K*_a_ of indomethacin is at around 4.5.^40^ An isosbestic point at 260 nm was identified between the ionized and the neutral form of the drug.^41^ At this wavelength, the molar absorptivity is independent of the ionization state, and it was selected to quantify the drug concentration to avoid the influence of ionization as shown in ESI Fig. S2–S5.† indomethacin in solution of high pH (pH = 13) has shown a completely different absorbance profile, which is expected due to indomethacin being rapidly hydrolyzed at this pH (ESI Fig. S3B†).^42^

To confirm that indomethacin is stable in the solution tested in the phase solubility studies, and the interaction between the drug and amino acid does not produce large changes to the UV absorbance, the molar absorptivity of indomethacin in arginine, lysine, histidine solutions and aqueous solution at pH 10.3, 10 and 7.5 and 6.3, respectively are determined and shown in ESI Fig. S5.† The results have shown that the molar absorptivity of indomethacin at 260 nm was not influenced by changing pH from 6.3 to 10.3 in the presence of different amino acids. The ionized drug remains stable for the duration of the experiment (less than 24 hours) at the pH range of the basic amino acid solutions tested in this study. However, the drug was found to be gradually degraded after 24 hours in arginine and lysine solutions at pH 10.3 and 10, respectively, whilst with histidine, where the pH of the solution was 7.5, the drug was stable over a week.

### pH-Solubility of indomethacin

Table 2 shows the intrinsic solubility (*S*_0_) of the unionized indomethacin and the total aqueous solubility (*S*) of the ionized and unionized forms at 25 °C and 30 °C.^43,44^ The solubilities obtained were used as a reference for comparison and understanding the drug–amino acid complexes purposes.

**Table 2:** Indomethacin intrinsic solubility (*S*_0_) and aqueous solubility (*S*) at different temperature 25 ± 2 °C and 30 ± 2 °C as mean concentrations ± SD (*n* = 3)

Crystalline phase	Temperature (°C)	Intrinsic solubility (mM)	Aqueous solubility (mM)
IND	25	(0.95 ± 0.1) × 10^−3^	(64.25 ± 0.5) × 10^−3^
	30	(1.18 ± 0.2) × 10^−3^	(81.64 ± 1.2) × 10^−3^

### Phase solubility studies

**Fig. 2:**
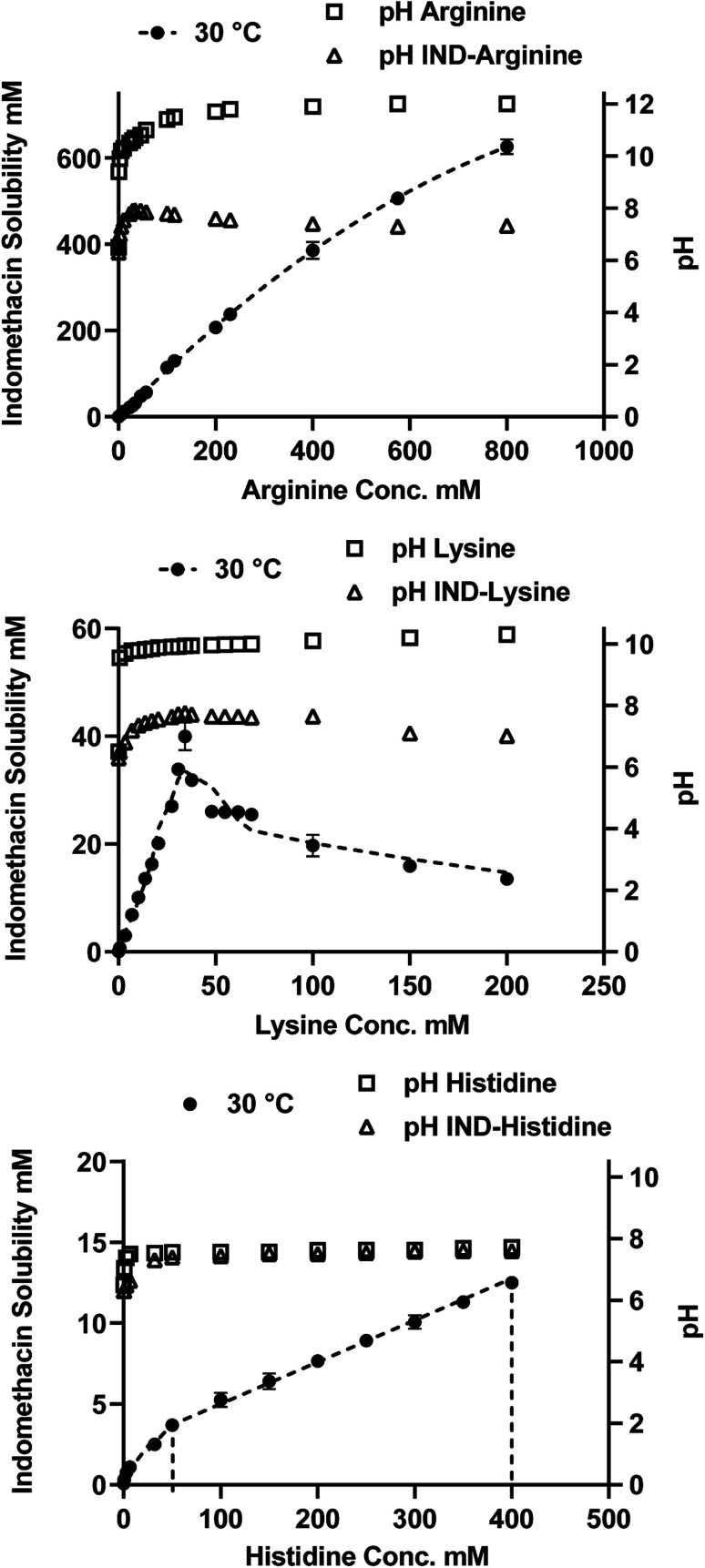
Phase solubility diagrams of indomethacin in aqueous arginine (a), lysine (b) and histidine (c) amino acid solutions at temperature 30 °C with pH measurements as mean concentration ± SD (*n* = 3), highlighting the linear part between indomethacin and histidine.

Indomethacin is expected to show higher solubility in solutions of arginine and lysine than in solution of histidine. This is due to the stronger basic guanidine group in arginine and amine in lysine, reaching around pH 12 and pH 10.3 respectively in the pure amino acid solutions, as compared to the less basic imidazole group in histidine, reaching pH 7.6 in the pure amino acid solution as shown in Fig. 2. The higher pH indicates that the solubility improvement in the former two solutions is predominantly due to the stronger ionic interactions between the carboxylate group of indomethacin and the ammonium group of the amino acids through the formation of salt.

#### IND–ARG complexes

**Fig. 3:**
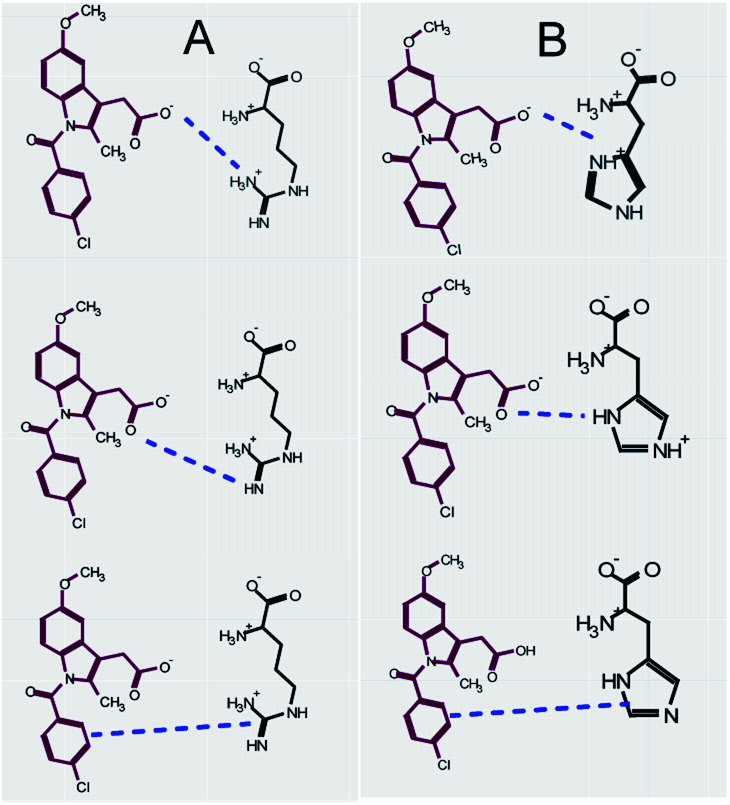
Solution state iteration between indomethacin and arginine (A), and indomethacin and histidine (B).

The phase solubility diagram of indomethacin in the presence of different concentrations of arginine amino acid is shown in Fig. 2(a). Below 250 mM of arginine, the solubility of indomethacin was increased linearly, which is described as an A_L_-type PS diagram according to Higuchi and Connors with a 0.92 : 1 drug–arginine mole ratio as shown in Table 3. After adding indomethacin to the arginine solution, the pH was dropped from between 9–12 to 6–8 (depending on the concentration of arginine) as shown in Fig. 2(a), confirming that neutralization has occurred. Molecules are interacting mostly through ionic interactions. The result shows that the ionic interaction between the carboxylate moiety of indomethacin and the guanidinium of arginine dominates at arginine concentrations of below 250 mM. At above 250 mM, however, the solubility diagram of indomethacin has shown a negative deviation from linearity (A_N_-type PS diagram) with little changes in the pH against an increasing drug and amino acid concentration, suggesting that the ionic strength of aqueous complexes might be affected.^45^ It indicates that the ionic interactions were interrupted by either other non-ionic interactions or self-association of arginine followed by precipitation at high concentrations, which has been previously reported.^46^ Further investigation on the undissolved drug/precipitation was conducted to fully characterize and understand the complexations at high concentrations of arginine. The solid phase is expected to mainly consist of an excess solid drug after equilibration but, potential, also contained the precipitation of arginine after the addition of the drug. The FTIR results showed when subtracting the solid precipitate spectrum from pure indomethacin, no remainder peaks were detected, suggesting that only the excess drug was detected in the solid phase and arginine did not self-association and precipitate at high concentration. The loss of linearity between drug and arginine high concentration is therefore most likely due to the interruption by non-ionic interactions such as H-bonding or H–π rather than self-association, which are competing with the ionic interactions as shown in Fig. 3.

**Table 3:** The linear equation (slop and intercept) with *R* square of phase solubility diagrams of indomethacin–amino acids and the complexation efficiency (CE)

Analytical parameter	Arginine	Lysine	Histidine
Conc. (mM)	(Up to 35 mM)	(Up to 20 mM)	(50 mM to 400 mM)
Linear equation	0.921*X* + 0.082	0.973*X* + 0.082	0.030*X* + 0.082
*R* Square	0.998	0.999	0.999
Complexation efficiency (CE)	11	36	0.03

#### IND–LYS complexes

**Fig. 4:**
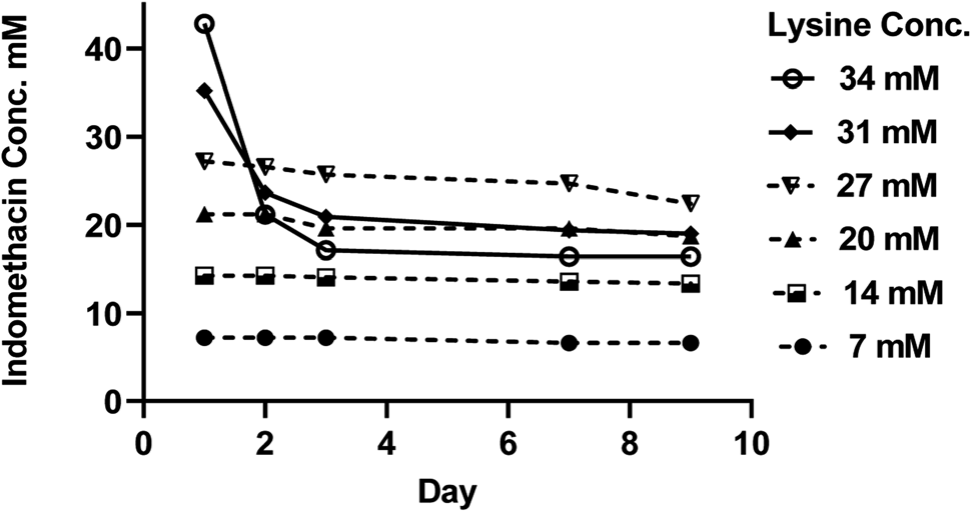
The stability of indomethacin in different concentration of lysine aqueous solution examined for ten days as mean concentration ± SD (*n* = 3).

When indomethacin was added to the lysine solutions, a complex curvature phase solubility diagram, which is regarded as a B_s_-type PS, was observed as shown in Fig. 2(b). This indicates that indomethacin–lysine complexes or self-associated lysine with limited solubility in the aqueous solution have formed at high concentrations of lysine. The solubility of indomethacin was increased proportionally as a function of lysine concentration up to 27 mM, producing a 0.97 : 1 drug : amino acid mole ratio as shown in Table 3, indicating that at relatively low lysine concentrations, ionic interaction between the indomethacin carboxylate and the ammonium ion of lysine dominates. The drop in pH of the drug–amino acid solution of 6–8 as shown in Fig. 2(b), confirms the neutralization has occurred. However, Fig. 4 shows that while the concentration of indomethacin appears stable at lysine concentrations below 27 mM, solutions with lysine concentration between 27 and 37 mM has dropped after 1 day of storage, suggesting that the solutions were supersaturated and the dissolved drug was partially precipitated. In the plateau region in the type-B_s_ PS diagram, the indomethacin solubility was constant even when the lysine concentration was increased, indicating that the indomethacin–lysine complexes have reached their limit of solubility. Further increase of lysine concentration to above 75 mM has shown the solubility of indomethacin decreased, indicating that a new interaction, potentially due to polymerization of lysine to poly-l-lysine and the precipitation of the self-aggregated poly-l-lysine and indomethacin.^47^ At lysine concentration above 200 mM, the drug–amino acid mixture started to thicken and turned into a white solid as a result of the extensive polymerization of lysine and the resulting interactions between poly-l-lysine and the drug.^48^ This indicates that more than one complex species be formed between indomethacin and lysine in the phase solubility system.

**Fig. 5:**
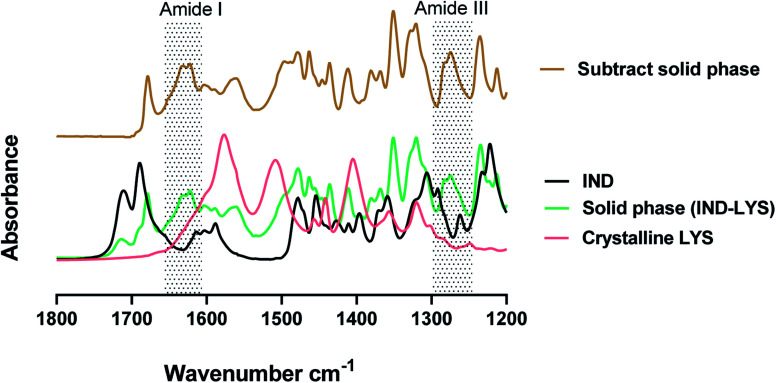
FTIR spectra of pure γ-indomethacin (IND) and crystalline lysine (LYS) as received, the solid phase indomethacin in 200 mM lysine (solid phase IND–LYS) and subtract spectrum of the pure indomethacin from the solid phase indomethacin in 200 mM lysine (subtract solid spectrum).

The solid phase from the lysine solutions (27 mM and 200 mM) was investigated *via* FTIR. The result of the subtracted spectrum of the solid phase in the <27 mM lysine solution from the pure indomethacin spectrum shows no remainder peaks. This indicates that lysine did not precipitate and remained in solution at low concentrations of lysine. Fig. 5 shows the FTIR spectra of pure γ-indomethacin, the crystalline lysine as received, the spectrum of the solid phase of the indomethacin in lysine (200 mM) and the subtract spectrum of the solid phase from pure indomethacin. In contrary to the spectrum of the solid phase from the 27 mM solution, the spectrum of the solid phase from the 200 mM solution, after subtracting the spectrum of the pure drug, has shown peaks at 1274 cm^−1^ and 1283 cm^−1^, 1560 cm^−1^, 1622 cm^−1^ and 1633 cm^−1^. These peaks are not present in either indomethacin or lysine and can be assigned as the amide III, II and I of poly-l-lysine providing the evidence that lysine was polymerized to poly-l-lysine and precipitated at high lysine concentration.^49–51^ The results show that the solid precipitate was a mixture of solute (the excess crystalline indomethacin) and poly-l-lysine. Continuous stirring or increasing the indomethacin–electrolyte concentration to the solution could be the reasons for hastening the polymerization of lysine.

#### IND–HIS complexes

The phase solubility diagram of indomethacin in histidine solutions at 30 °C and 45 °C is shown in Fig. 2(c). The improvement in the indomethacin solubility in the presence of histidine is significantly less than with arginine and lysine. At low (*e.g.* <5 mM) concentration of histidine, a 0.15 : 1 drug : amino acid mole ratio can be observed and the ratio decreases with increasing histidine concentration. At high concentration (>50 mM), however, the indomethacin solubility shows a linear improvement, highlighted in Fig. 2(c). The limited solubility improvement is expected as histidine is a weaker base. The pH of the drug–amino acid solutions (Fig. 2(c)) is measured at ∼7.5 in most concentrations of histidine, except for the lowest concentrations. Knowing that the pKa of the imidazole group is ∼6, the ring is primarily in the neutral form at pH 7.5.^52^ This suggests that the ionic interaction has a relatively limited effect and may be easily disrupted by other molecular interactions, which explains the reduced improvement in indomethacin solubility with the increasing histidine concentration at <50 mM. Other molecular interactions may result in self-aggregates or non-ionic complexations through CH–π and π–π interactions or hydrogen bonds between the imidazole group of histidine and the aromatic rings of indomethacin as shown in Fig. 3.^53^ The solid phase after equilibration in the indomethacin–histidine aqueous solution (300 mM) was investigated *via* FTIR. The same findings with ARG were observed with HIS, suggesting that histidine was stable in the solution and does not precipitate.

The solubilizing efficiency of these amino acids was evaluated by determining the complexation efficiency from the slop of the A-type drug-amino acid PS diagrams.^36^Table 3 shows the values of complexation efficiency calculated by eqn (1). Lysine shows the highest solubilization efficiency of the three basic amino acids tested at the linear drug–amino acid PS diagrams. At high concertation amino acid, however, arginine shows higher potential for the solubilization effect than others due to the better stability in the solution. Overall, the solubilization effect of amino acids to enhance the solubility of class II indomethacin is decreased as a function of concentration potentially due to the disruption of the ionic interaction by either self-association or non-ionic interactions at high amino acid concentrations.

### Hydrotropic effect

**Fig. 6:**
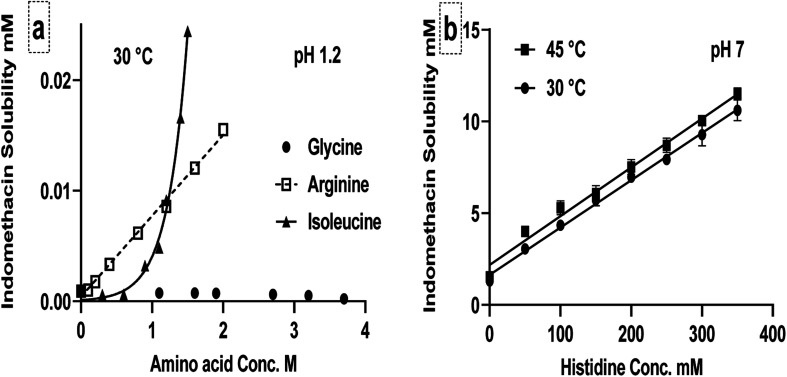
Equilibrium solubility of indomethacin in arginine, glycine and isoleucine aqueous solutions controlled at pH 1.2 at 30 °C (a) and in aqueous histidine solution controlled at pH 7 temperature 30 °C and 45 °C (b) as mean concentration ± SD (*n* = 3).

It has been shown in Fig. 2 that indomethacin solubility was improved mainly due to ionic interactions with basic amino acids; the result in the deviation from the linear relationship also shown that other molecular interactions exist. Non-ionic interactions, such as hydrotropic effect that can improve the solubility of drugs even when they are not ionized, are less explored. This is important because non-ionized molecules have better cell membrane permeability, thus many oral drugs may be specifically designed to be non-ionizable. We have demonstrated that neutral amino acids can act as hydrotropes for ionized indomethacin.^30^ To demonstrate the potential of charged amino acids as hydrotropes for the unionized form of indomethacin in the presence of different concentrations of arginine, isoleucine and glycine at pH 1.2 are obtained and the results are shown in Fig. 6(a). At pH 1.2, indomethacin is mostly in the unionized form and amino acids are positively charged as shown in ESI Fig. S3A.† The solubility of the unionized indomethacin had improved by almost 11-fold in 1.6 M arginine solution and 25-fold in 1.5 M isoleucine solution compared to the intrinsic solubility in water, whereas the indomethacin solubility did not change with the same concentration of glycine. These improvements are greater than the hydrotropic effect observed from neutral amino acids. The reason for the improvement may be caused by inductive forces such as ion-induced dipole interaction and dipole-induced dipole interaction between polar arginine side chain and nonpolar indomethacin.^54^ The relationship between indomethacin and arginine was linear, suggesting that it was due to the saturation effect of complexes. With isoleucine, however, the indomethacin solubility was improved following an exponential relationship, suggesting self-association was formed at high concentration due to amphipathic nature of isoleucine in acidic pH.^55^ Positively charged glycine did not improve the solubility perhaps due to the lack of hydrophobic moiety and the small molecular size. This concludes that hydrotropic effect is not limited to neutral (uncharged) amino acids, but also with charged amino acids as shown in the current study.

Fig. 6(b) shows the solubility of ionized indomethacin in histidine solution at pH 7 (the isoelectric point of histidine), where the overall change of the molecule is neutral. This was conducted to demonstrate the non-ionic interactions between neutral histidine and ionized indomethacin. The solubility of indomethacin was improved by almost 8-fold in 0.35 M histidine solutions at pH 7 compared to the aqueous solubility in the same pH but without histidine, confirming the involvement of non-ionic interactions on the improvement of drug solubility. This improvement was linear, similar to tryptophan,^30^ indicating that both histidine and tryptophan have the strongest hydrotropic effect among the amino acids studied.

### Molecular characterisation of freeze-dried indomethacin–amino acid using UV-VIS, FTIR, DSC and TGA

UV-VIS, FTIR, DSC and TGA were conducted for the freeze-dried drug-amino acid systems to characterize the solid state such as drug content, phase transitions, chemical and thermal stability, and the potential interactions.

#### UV-VIS

The indomethacin content in freeze-dried mixtures was determined *via* UV-VIS method using eqn (2). It has shown that 62.1% ± 1.8 and 63.1% ± 2.9 of indomethacin were obtained in arginine and lysine, respectively (equivalent to ∼1 : 1 mole ratio). With histidine, however, the drug content was found to be 22.1% ± 0.74. Moreover, indomethacin was chemically stable with all amino acids after freeze-drying as shown by the stable peak position at 320 nm.^42^

#### FTIR

**Fig. 7:**
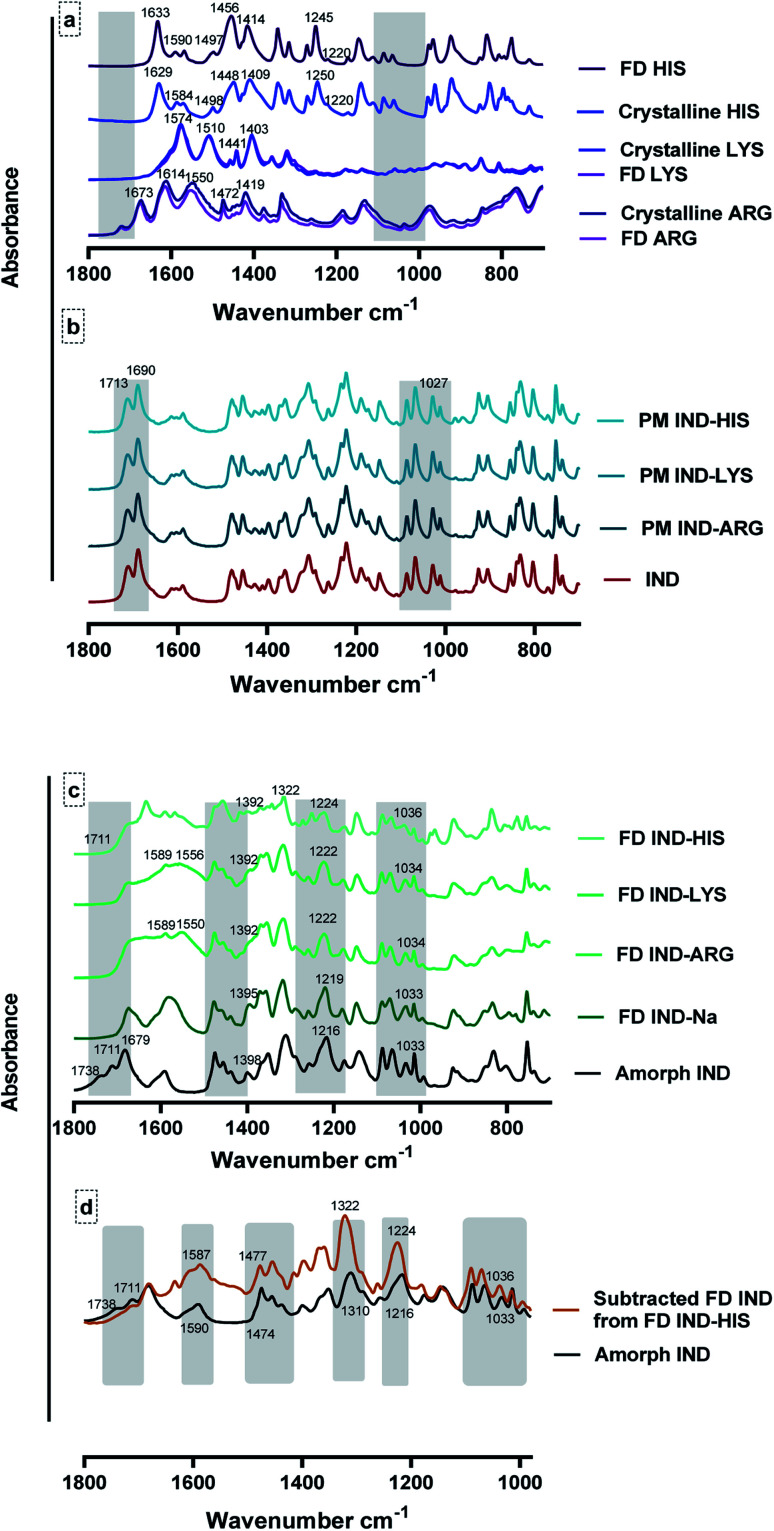
FTIR spectra of (a) pure crystalline amino acids as received and freeze dried (FD) from aqueous solution, including arginine (ARG), lysine (LYS) and histidine (HIS). (b) γ crystalline indomethacin (IND) as received and physical mixture (PM) of indomethacin–basic amino acids (1 : 1) molar ratio. (c) Amorphous indomethacin (amorphous IND) obtained by quench cooling and freeze-dried saturated indomethacin–arginine (FD IND–ARG), FD IND–LYS, FD IND–HIS and FD IND–sodium (Na) from aqueous solubility studies *n* = 3. (d) Comparison between amorphous indomethacin (Amorph IND) and subtracted FD IND from FD IND–HIS, highlighting the main peaks.

Fig. 7(a) and (b) shows the FTIR spectra of pure crystalline amino acids (arginine, lysine and histidine) and the γ form of drug as received, freeze-dried from aqueous solutions and the physical mixture of drug–amino acids (1 : 1) molar ratio. The freeze-dried histidine spectrum shows a slight shift in peak positions at 1633 cm^−1^, 1590 cm^−1^, 1497 cm^−1^, 1456 cm^−1^, 1414 cm^−1^ and 1245 cm^−1^ compared to crystalline histidine, suggesting that histidine might be partially amorphized during the freeze-drying cycle. With arginine and lysine, there are no differences between the crystalline and the freeze-dried amino acid, showing that the freeze-drying process does not produce an amorphous form for these compounds.^56^

Fig. 7(c) shows the spectra of amorphous indomethacin and the freeze-dried solution of the indomethacin–amino acid mixtures and the freeze-dried indomethacin–sodium salt which was included for comparison purposes.^57,58^ The spectra of the freeze-dried drug–amino acid complexes show clear differences when compared to the individual freeze-dried compounds. The most significant changes can be observed in the spectral region of 1800 cm^−1^ to 700 cm^−1^. The freeze-dried indomethacin–amino acids have shown a loss of the carboxylic acid bands at 1711 cm^−1^ and 1738 cm^−1^ and a gain in the asymmetric and symmetric COO^−^ stretching bands at 1589 cm^−1^ and a shoulder at 1414 cm^−1^, respectively, when compared to the crystalline or amorphous indomethacin as shown in Fig. 7(b) and (c). This is due to the dissociation of the COOH group producing a similar spectrum as the freeze-dried sodium–indomethacin salt. The peak at 1550 cm^−1^ in the freeze-dried indomethacin–arginine salt can be assigned to the *ν*(NH_3_)^+^ arginine side chain confirming the ionic state of both the drug and the amino acids.^56,59^ There are small but reproducible shifts of the 1216 cm^−1^ peak, corresponding to benzene–CH bending in amorphous indomethacin, to 1219 cm^−1^ in the freeze-dried indomethacin–sodium salt and to 1222 cm^−1^ in the freeze-dried indomethacin–arginine salt.^14^ Moreover, the peak at 1033 cm^−1^ in amorphous indomethacin and freeze-dried indomethacin–sodium salt, corresponding to the benzene ring was shifted to 1034 cm^−1^ in the freeze-dried indomethacin–arginine salt.^59^ These small but reproducible shifts suggest the involvement of non-ionic interactions in the complexations as discussed in the phase solubility studies. Another apparent shift could be found at 1398 cm^−1^ for the amorphous indomethacin indole ring deformation. This band appeared at 1395 cm^−1^ in the freeze-dried indomethacin–sodium salt and being a shoulder at lower wavenumber at 1392 cm^−1^ in the freeze-dried indomethacin–arginine salt, strongly suggesting non-ionic interaction between indomethacin and arginine.^14^ Furthermore, the plateau-like feature at ∼1650 cm^−1^ region has been observed in the freeze-dried indomethacin–arginine spectrum but not in the indomethacin sodium salt spectrum, confirming that complex H-bonds are involved in the drug–arginine interactions. The four characteristic peaks between 1100 cm^−1^ and 950 cm^−1^, have shown a similar pattern to the amorphous indomethacin reference spectrum but different from the crystalline indomethacin spectrum, confirmed that freeze-dried arginine-indomethacin and indomethacin sodium salt were amorphous.^58^ A similar finding was observed in all complexes of freeze-dried indomethacin–lysine and indomethacin–histidine as shown in Fig. 7(c). However, the spectrum of the freeze-dried indomethacin–histidine was mainly contributed by histidine as a result of the limited solubility of indomethacin in histidine solution. To focus on the spectrum of the drug, Fig. 7(d) shows the comparison between amorphous indomethacin and the freeze-dried indomethacin–histidine with the freeze-dried histidine subtracted. The subtracted spectrum shows significant differences compared to individual amorphous indomethacin. The most significant observation made in the subtracted freeze-dried indomethacin–histidine spectrum is the carboxylic acid bands of indomethacin at 1711 cm^−1^ and 1738 cm^−1^, which were not observed in the freeze-dried indomethacin–arginine and lysine, indicating that instead of the salt form, the neutral form of the drug was obtained in the freeze-dried indomethacin–histidine.^59^ Furthermore, the plateau-like feature at 1650 cm^−1^ region in the subtract freeze-dried indomethacin suggests that H-bonds may be involved. Another apparent shift could be observed at 1590 cm^−1^ for indole ring stretching in the amorphous indomethacin. This band appeared in the subtract freeze-dried indomethacin at around 1587 cm^−1^. Moreover, significant bands were shifted from 1474 cm^−1^, 1310 cm^−1^,1216 cm^−1^ and 1033 cm^−1^ in the amorphous indomethacin, corresponding to CH_3_ deformations, CH_2_ wagging coupled with phenyl–CH deformations and benzene–CH bending and benzene ring, respectively to 1477 cm^−1^, 1322 cm^−1^, 1224 cm^−1^ and 1036 cm^−1^, in the subtracted freeze-dried indomethacin.^59^ These shifts confirm the involvement of the non-ionic interactions such as H–π and π–π stacking at the aromatic moieties between histidine and drug.^53^ In summary, the changes observed for the indomethacin in the freeze-dried indomethacin–amino acids complexes indicate the involvement of multiple interactions between indomethacin and basic amino acids. These interactions are most likely to consist of ion-pair as the major contributor. H-bonding, CH–π and π–π interactions with the hydroxyl, aliphatic, aromatic and amide groups are also evident from the shifts observed in their respective spectral bands.

#### DSC

**Fig. 8:**
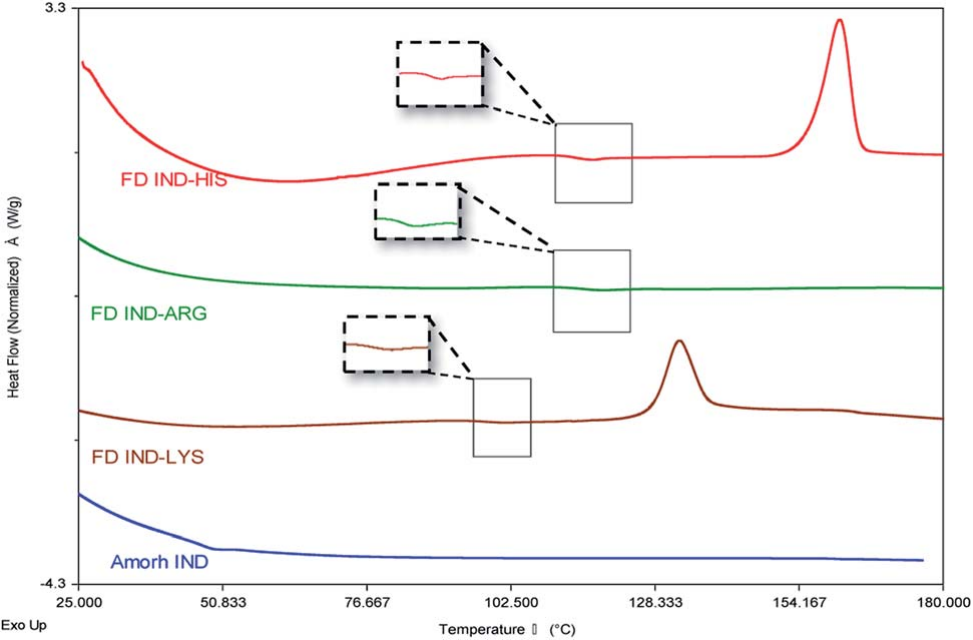
DSC thermogram of amorphous indomethacin (Amorph IND) and freeze-dried complexations drug–amino acids including arginine, lysine and histidine at heating rate of 5 °C min^−1^, highlighting the glass transition temperatures.

The results showing the phase transitions of pure drug, amino acids or their mixtures (prepared by freeze-drying or physical mixing) are shown in Fig. 8, ESI Fig. S6, S7† and Table 4. Fig. S6 and S7† show that pure indomethacin and amino acids did not produce amorphous form by freeze-drying, with only the *T*_g_ of the drug (42.1 ± 0.5 °C) was obtained *via* melt-quenching. Fig. 8 also shows that the *T*_g_s of all freeze-dried drug-amino acid systems have increased compared to the amorphous drug. A single *T*_g_ is found in all cases indicates that the obtained systems were homogeneous amorphous mixtures. Elevated *T*_g_s are desirable for improving physical stability of the amorphous form.^39^ The *T*_g_ endotherms of indomethacin–lysine and indomethacin–histidine systems were followed by a re-crystallization exotherm and then a melting event at high temperature, suggesting this was due to the re-crystallization of amino acids (data not shown). However, this re-crystallization exotherm was not observed with indomethacin–arginine, suggesting it is the most stable system studied in this work. Furthermore, all freeze-dried systems show no melting peak at 160 °C, confirming the drug remained amorphous.

**Table 4:** The experimental (exp.), literature (lit.) and the Gordon–Taylor glass transitions (*T*_g_s) of pure and freeze-dried indomethacin drug and basic amino acids arginine, lysine and histidine; the experimental and literature melting temperatures; preparation method and molar ratio (*n* = 3)

Sample Content	Molar ratio	Method	*T*_g_ (°C), exp.	*T*_g_ (°C), lit.	*T*_g_ (°C), Gordon–Taylor	*T*_m_ (°C), exp.	Tm (°C), lit.
IND		Melt quench	42.1 ± 0.5	42.0 (ref. 60)	42.1	160.0 ± 0.2	161.0 (ref. 61)
ARG		Pure		55.0;^62,63^ 34.0;^10^ 18.4 (ref. 64)	55.0	232.4 ± 1.7	236.1 (ref. 65)
LYS		Pure		68.0 (ref. 63)	68.0	212.1 ± 0.2	212.0 (ref. 65)
HIS		Pure		37.0 (ref. 63)	37.0	275.4 ± 0.2	280.0 (ref. 65)
IND–ARG	1 : 1	FD	116.2 ± 1.1		45.6		
IND–LYS	1 : 1	FD	100.1 ± 1.5		47.5		
IND–HIS	0.15 : 1	FD	116.2 ± 0.9		38.0		

It is possible to identify specific interactions between drug and amino acids through the experimental *T*_g_s for the freeze-dried amorphous mixtures. The *T*_g_s of the individual amorphous compounds and the *T*_g_s calculated by the Gordon–Taylor equation (eqn (3) and (4)) are shown in Table 4. The large deviations from the Gordon–Taylor equation indicate strong interactions between compounds due to the formation of salt and the non-ionic effects.^39^ This is in agreement with the results obtained in the solubility and FTIR studies.

#### TGA

The TGA results show there is a 5% weight loss at below 100 °C in the crystalline arginine and lysine, which corresponds to the residual water in the hygroscopic compounds. However, this was not observed with histidine as shown in ESI Fig. S8.† ^66^ Moreover, the TGA thermograms of pure crystalline indomethacin and amino acids showed major weight loss after *T*_m_ with rapid decomposition.^38^ However, all freeze-dried drug–amino acids complexes show elevated thermal stability of the drug in presence of amino acids as there is a reduction in the total weight loss compared to the pure individual drug (ESI Fig. S10†). This concludes that amino acids can improve the thermal stability of drug when added as excipients.

In summary, basic amino acids (arginine, lysine and histidine) have been successfully used as counterion co-formers to produce the salt form of an acidic class II BCS drug (indomethacin). These non-toxic small molecule excipients have shown advantages of enhancing solubility and stability of amorphous drugs. However, non-ionic interactions between basic amino acids and class II drugs have not been clearly identified, which is important as these interactions are less dependent on the pH of the solution and thus may help to overcome issues such as disproportionation during storage and dissolution.

In the current study, the nature of complexations between compounds was found to play major role in the production of consistent formulations. In the liquid state, ionic strength can be influenced by the amino acid concentration and consequently the electrostatic interaction between drug and amino acids. At low concentrations of arginine (<35 mM) and lysine (<20 mM), the ionic interaction between drug–amino acid dominates with a neglectable effect from other form of interactions. At higher concentrations, however, the results show that the ionic interaction was disturbed by either non-ionic interactions (arginine and histidine) or self-association (lysine). The potential non-ionic interactions involved include H-bonding, H–π and π–π stacking. The self-association was observed with lysine complexation by forming poly-l-lysine. The mole ratio between the indomethacin and arginine and lysine in water is almost ∼1 : 1. However, this was not observed with histidine due to the weaker basic side chain. At acidic pH, the solubility of the unionized form of the drug in amino acid solutions was improved with increasing concentration of amino acids. This indicates that the overall positively charged amino acids have hydrotropic effect, similar to our previous study on non-charged amino acids.^30^ In the solid state, the freeze-dried supernatant of the amino acids–drug solution shows successful production of co-amorphous formulations in all three amino acids with a mole ratio of almost ∼1 : 1 for indomethacin–arginine and lysine, and ∼1 : 7 for indomethacin–histidine. All systems exhibit an elevated glass temperature compared to the individual compounds, as expected due to the strong ionic attraction with an additive effect of the non-ionic interactions including π–π stacking, H-bonding and CH–π.

## Conclusion

The mechanism of drug–basic amino acid complexations, with indomethacin as the model drug, was investigated by studying the phase solubility diagrams, FTIR measurement of the solid phase and the combined FTIR/UV/DSC/TGA analysis of the freeze-dried supernatant of the solution. The results have confirmed that non-ionic interactions are involved, which disrupted the ionic interactions, producing a negative deviation from linearity on the phase solubility diagram and reducing the solubilization effect of histidine, lysine and arginine. Particularly with lysine, the solubilization was disrupted by polymerizing causing a dramatic drop in indomethacin solubility at high (>27 mM) lysine concentrations. In acidic pH, where the drug is neutral, the amino acids have shown to be able to enhance the solubility without the ionic interactions. In the study of the freeze-dried supernatant, results have confirmed that the ion-pairing was the major interaction with some contributions from the weaker non-ionic interactions such as H-bonds, H–π and π–π.

## Author contributions

MSA: conceptualization; data curation; formal analysis; funding acquisition; investigation; methodology; validation; visualization; roles/writing – original draft. PGR: conceptualization; methodology; analysis; validation; writing – review & editing. KLAC: conceptualization; formal analysis; methodology; project administration; investigation; resources; software; supervision; validation; writing – review & editing.

## Conflicts of interest

The authors declare no conflict of interest.

## Supplementary Material

RA-012-D2RA02870K-s001
