# Supplementary material for: Mechanistic study of the solubilization effect of basic amino acids on a poorly water-soluble drug
Source: RSC Adv. 2022 Jun 29;12(30):19040–53. doi: 10.1039/d2ra02870k (PMC9240925; doi:10.1039/d2ra02870k)
Supplement: RA-012-D2RA02870K-s001 [file RA-012-D2RA02870K-s001.pdf]

Electronic Supplementary Material (ESI) for RSC Advances.
This journal is © The Royal Society of Chemistry 2022

Supplementary data

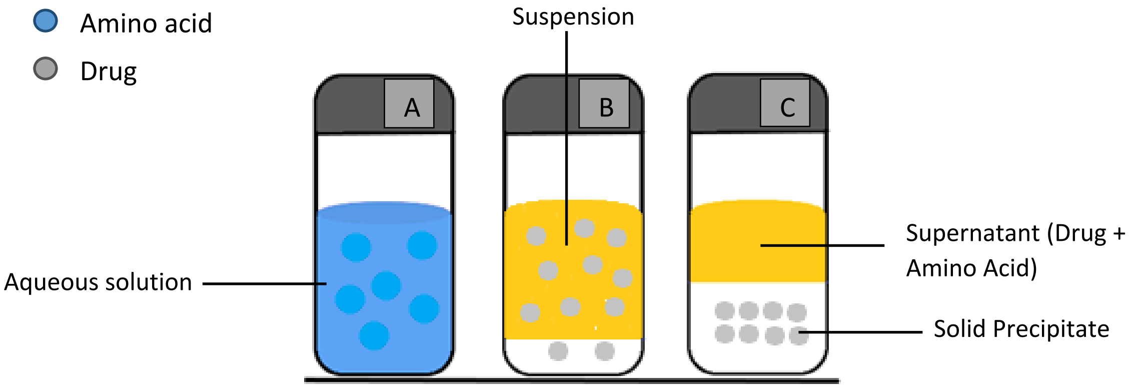

Figure S1: Exemplary of aqueous solution of dissolved amino acid (A); solution A with excess amount of indomethacin (B); solution B after centrifuged into the liquid supernatant and solid precipitation factions (C).

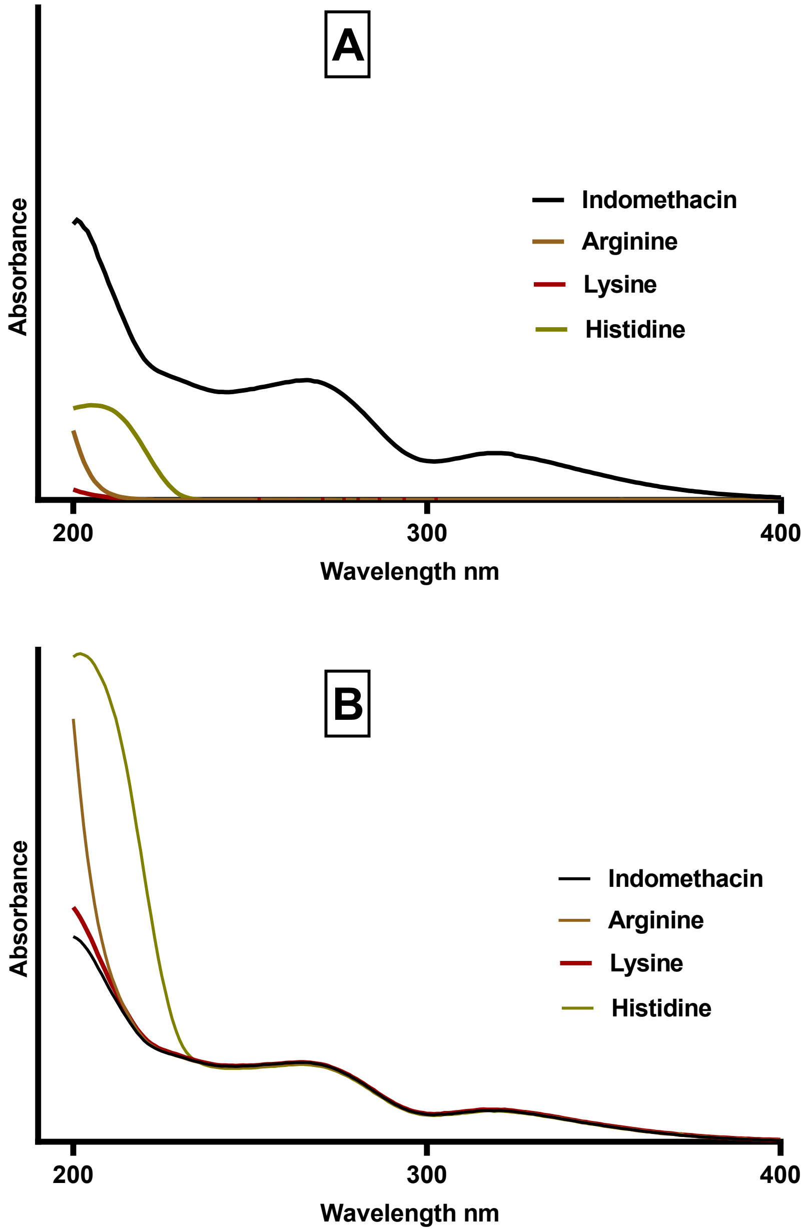

Figure S2: UV absorbance spectra of indomethacin and amino acid in water. (A) shows the spectra of pure ionized indomethacin and amino acids in water concentration 20 µg/mL. (B) shows the spectra of the ionized indomethacin (4 µg/mL) in amino acid (arginine, lysine and histidine) solution concentration of (20 µg/mL).

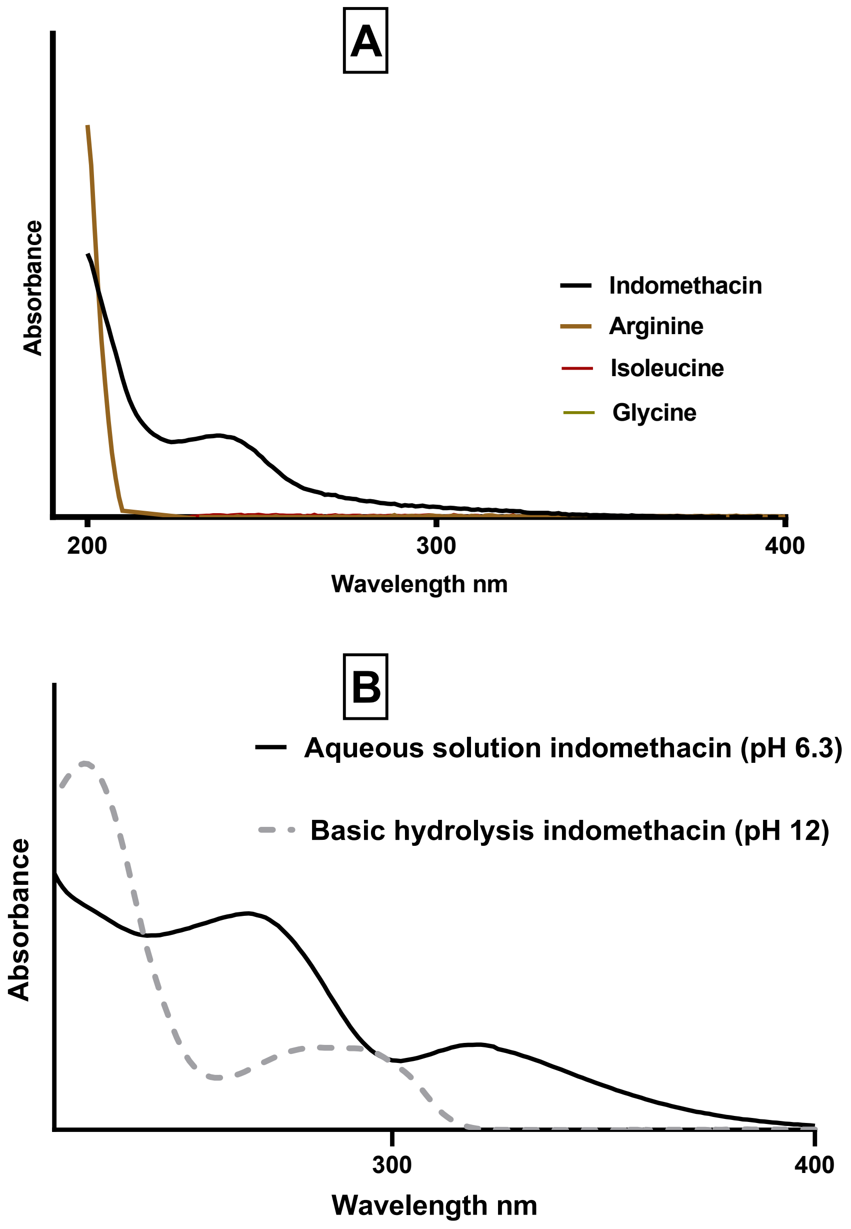

Figure S3: UV absorbance spectra of indomethacin and amino acids. (A) shows the spectra of pure unionized indomethacin and positively charged amino acids (arginine, glycine and isoleucine) at 0.5 μg/mL and 20 μg/mL in pH 1.2, respectively. (B) shows the spectra of the hydrolysed indomethacin in pH 12 and the unhydrolyzed ionized form of indomethacin in water pH 6.3.

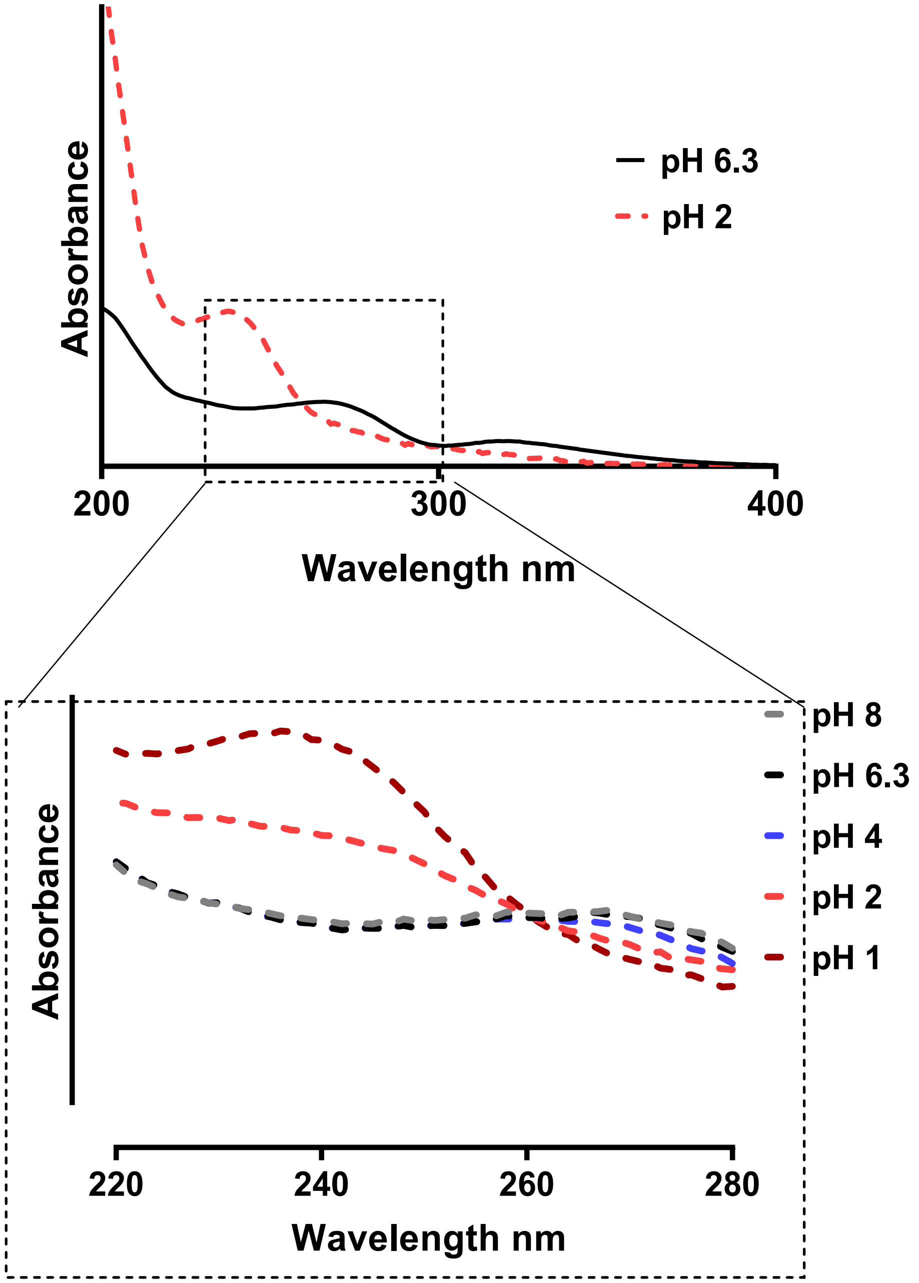

Figure S4: UV absorbance spectra of indomethacin in pH range 1-8, highlighting the isosbestic point that is used for the quantification the drug in ionized or unionized forms.

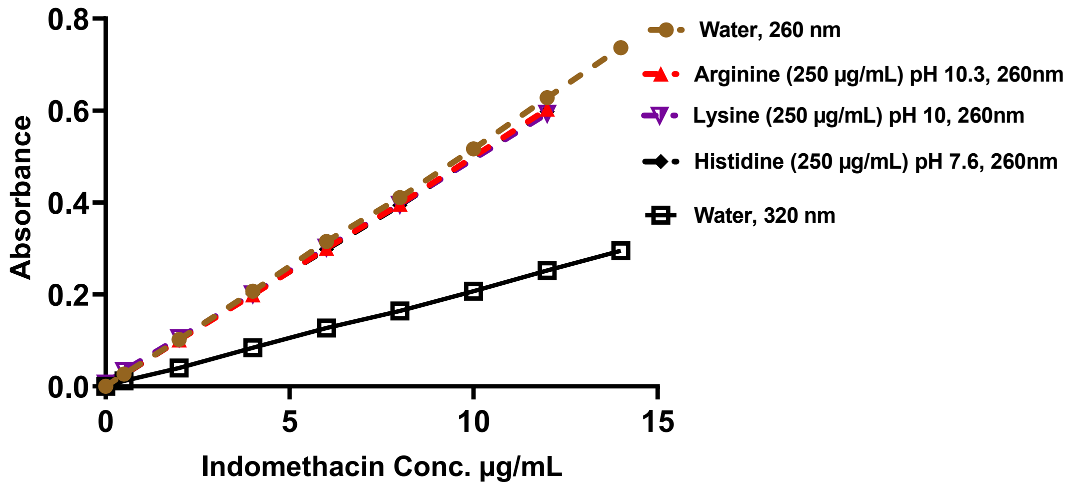

Parameter	Analytical Data				
Solution	Water		Arginine	Lysine	Histidine
Wavelength nm	320	260	260	260	260
Linearity Range (µg/mL)	0.5-14			0-12	
Slope	0.0209	0.0522	0.0496	0.0489	0.0492
Correlation Coefficient (R^2)	0.9999	0.9999	0.9999	0.9999	0.9999
Limit of Detection LOD (µg/mL)	0.47	0.42	0.27	0.20	0.24
Limit 0f Quantification LOQ (µg/ml)	1.41	1.26	0.82	0.61	0.71
Molar absorptivity (mol-1 cm-1)	7.48	18.4	17.90	17.8	17.8

Figure S5: Calibration curves of indomethacin in water, arginine solution, lysine and histidine (pH 6.3, 10.3, 10 and 7.6, respectively) obtained at 320 nm and 260 nm (the isosbestic point) and UV validation.

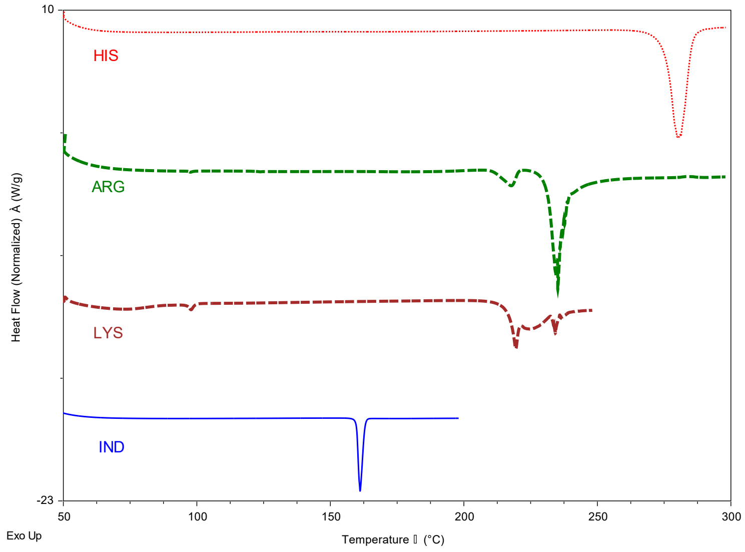

Figure S6: DSC thermogram of crystalline indomethacin (IND) and L-arginine (ARG), L-lysine (LYS) and L-histidine (HIS) as received at a heating rate of 5 °C /min.

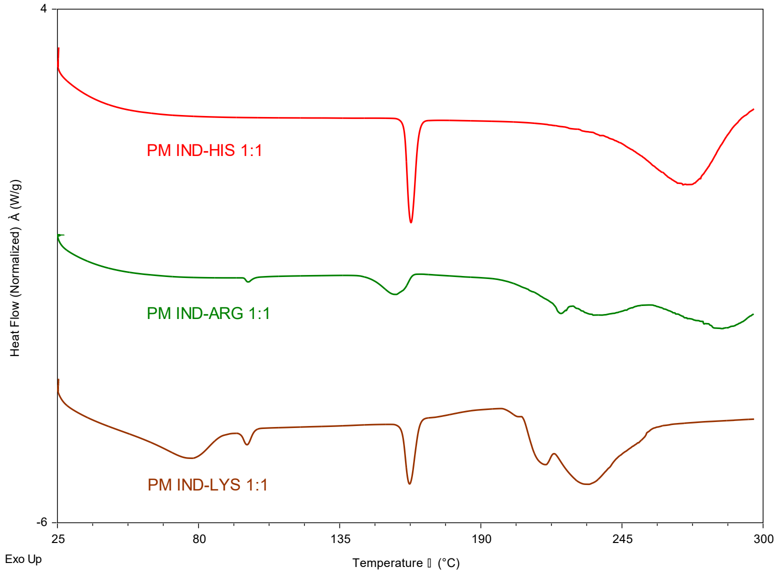

Figure S7: DSC thermogram of physical mixtures of indomethacin (IND)- amino acids including arginine (ARG), lysine (LYS) and histidine (HIS) at 1:1 molar ratio at a heating rate of 5 °C /min.

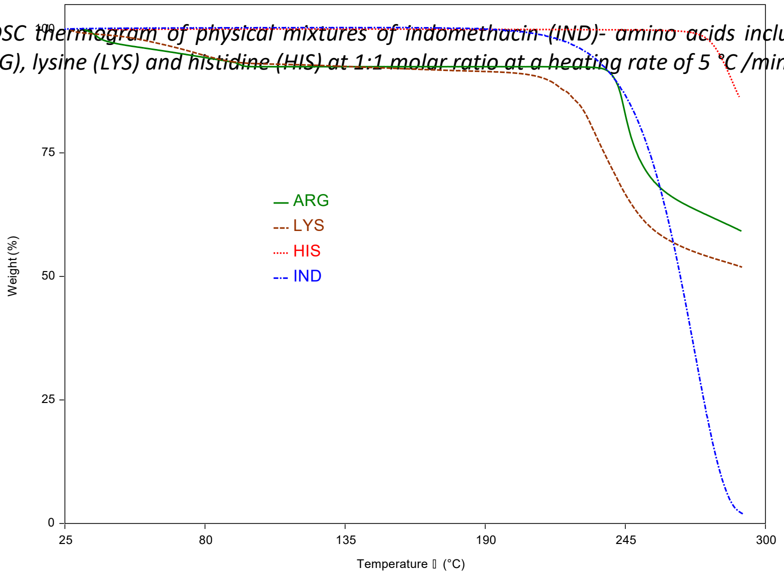

Figure S8: TGA thermogram of pure gamma for of indomethacin (IND), arginine (ARG), lysine (LYS) and histidine (HIS) as received, heated from room temperature to 300 °C, at a heating rate of 10 °C/min

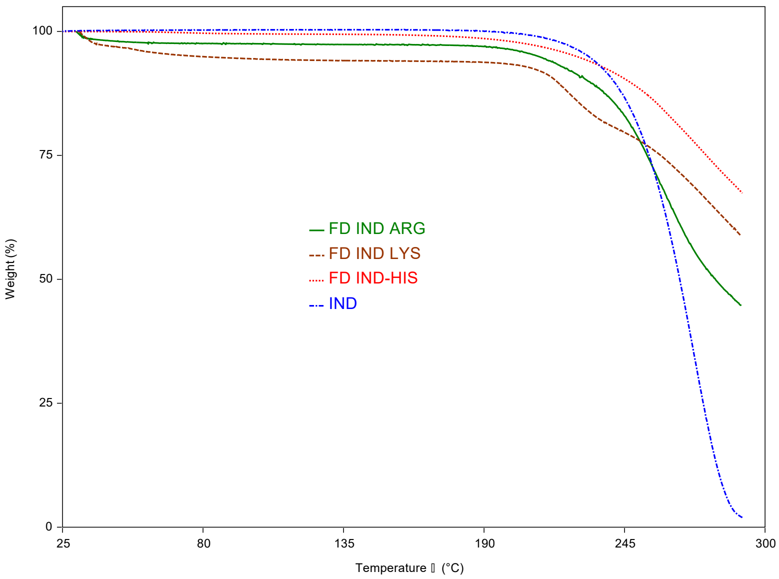

Figure S9: TGA thermogram of pure gamma indomethacin (IND) as received and physical mixtures indomethacin- amino acids including arginine (ARG), lysine (LYS) and histidine (HIS) at 1:1 molar ratio, heated from room temperature to 300 °C, at a heating rate of 10 °C/min

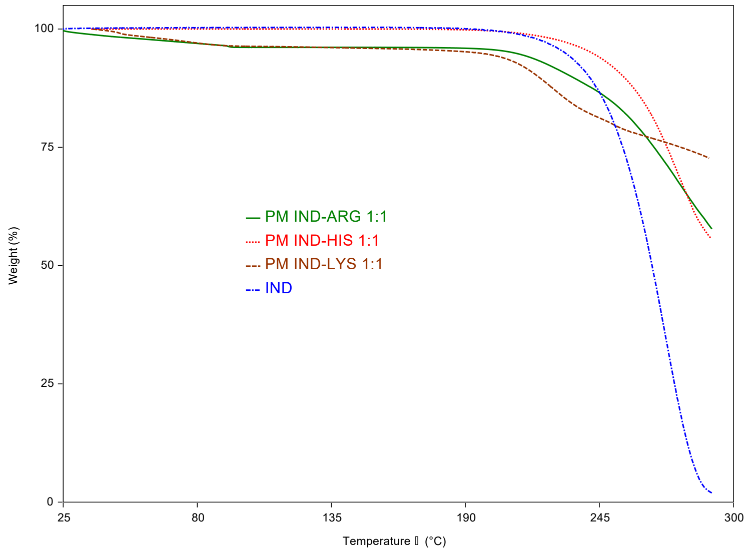

Figure S10: TGA thermogram of pure gamma indomethacin (IND) as received and freeze-dried indomethacin- amino acids including arginine (ARG), lysine (LYS) and histidine (HIS), heated from room temperature to 300 °C, at a heating rate of 10 °C/min
